# A trehalose 6-phosphate synthase gene of the hemocytes of the blue crab, *Callinectes sapidus*: cloning, the expression, its enzyme activity and relationship to hemolymph trehalose levels

**DOI:** 10.1186/1746-1448-4-18

**Published:** 2008-12-12

**Authors:** J Sook Chung

**Affiliations:** 1 Center of Marine Biotechnology, University of Maryland Biotechnology Institute, 701 E. Pratt Street, Columbus Center, Suite 236. Baltimore, MD 21202, USA

## Abstract

Trehalose in ectoderms functions in energy metabolism and protection in extreme environmental conditions. We structurally characterized trehalose 6-phosphate synthase (*TPS*) from hemocytes of the blue crab, *Callinectes sapidus*. *C. sapidus *Hemo *TPS *(*Cas*Hemo*TPS*), like insect *TPS*, encodes both *TPS *and trehalose phosphate phosphatase domains. Trehalose seems to be a major sugar, as it shows higher levels than does glucose in hemocytes and hemolymph. Increases in *HemoTPS *expression, TPS enzyme activity in hemocytes, and hemolymph trehalose levels were determined 24 h after lipopolysaccharide challenge, suggesting that both TPS and TPP domains of CasHemoTPS are active and functional. The *TPS *gene has a wide tissue distribution in *C. sapidus*, suggesting multiple biosynthetic sites. A correlation between TPS activity in hemocytes and hemolymph trehalose levels was found during the molt cycle. The current study provides the first evidence of presence of trehalose in hemocytes and *TPS *in tissues of *C. sapidus *and implicates its functional role in energy metabolism and physiological adaptation.

## Background

Trehalose, a non-reducing disaccharide is a primary energy source in prokaryotes, yeasts, plants, and invertebrates. The accumulation of trehalose in anhydrobioses of artemia, nematodes, and chironomids [1-3] implies a role in physiological and biochemical adaptations in extreme environmental conditions.

In insects, trehalose is the major hemolymph sugar that is exclusively synthesized in the fat body in which hypertrehalosemic hormone (HTH) positively regulates its production. In addition, flight, feeding, and parasitic infections in insects have been shown to produce hypertrehalosemia, i.e. an increase in trehalose in hemolymph [4-6]. These findings further support trehalose as an energy source and its involvement in physiological adaptation in insects.

Trehalose 6-phosphate synthase (*TPS*) is noted in insects as a fused gene that codes two functional domains in tandem: *TPS, a homolog of Ost A of Escherichia coli*, and trehalose 6-phophate phosphatase (*TPP*), a homolog of *Ost *B of *E. coli. Drosophila TPS *introduced into human HEK-293 cells increased hypoxia tolerance by which elevated trehalose reduced protein aggregation under hypoxia [2,7]. This result indicates two domains of *TPS *and *TPP *are active. However, a relationship between the level of *TPS *expression and TPS enzyme activity resulting in the increase in trehalose production has not been described in insects.

In contrast to hypertrehalosemic response under stress and during flight activity in insects, the increase in glucose level in hemolymph (i.e. hyperglycemia) of crustaceans has been described during their initial physiological adaptation to stressful environments [8-15]. Lipopolysaccharide (LPS) injection, an accepted method for mimicking a pathogen infection, also induced hyperglycemia through modulating the level of crustacean hyperglycemic hormone [13]. The glycogen present in many crustacean tissues, including hemocytes, is tacitly accepted as the source of this hyperglycemia. Previous reports of the involvement of trehalose in osmoregulation and cold adaptation in crustaceans [16,17] and the ubiquitous abundance of trehalose in insect hemolymph as an energy source and its protective roles under stress emphasize the importance of this molecule in invertebrates. Therefore, we investigated the presence of *TPS *gene and trehalose in the blue crab, *Callinectes sapidus*, the population of which has been drastically declining in the Chesapeake Bay [18], in order to better understand the role of this sugar in energy metabolism during molt cycles and physiological adaptation under stressful conditions. Particularly, in an attempt to define an adaptive role of trehalose in a different physiological status of *C. sapidus*, we challenged animals with LPS that generally induced the response of a pathogen infection as well as the stress response of hyperglycemia in crustaceans [13,15]. We demonstrated hypertrehalosemic and hyperglycemic responses by LPS injection into the animal that was accompanied by increases in *TPS *expression and TPS enzyme activity in hemocytes. A relationship between TPS activity in hemocytes and the level of hemolymph trehalose during a molt cycle was established.

## Results and discussion

### Phylogenetic tree analysis of multiple sequence alignments of *TPS *gene

**Figure 1:**
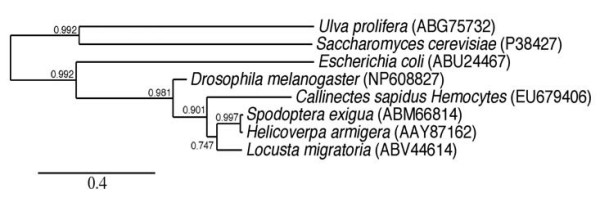
Phylogenetic tree analysis of deduced amino acids of TPS including *Callinectes sapidus *hemocytes (EU679406), *Spodoptera exigua *(ABM66814), *Helicoverpa armigera *(AAY87162), *Locusta migratoria *(ABV44614), *Drosophila melanogaster *(NP608827), *Escherichia coli *(ABU24467), *Saccharomyces cerevisiae *(P38427), and *Ulva prolifera *(ABG75732). The neighbor-joining tree was constructed and bootstrapped (1000 iterations) using Robust Phylogenetic Analysis for the Non-Specialist [19]. Bootstrap values are noted on the branch and the scale bar (= 0.4) represents fixed mutations per amino acid position.

*Cas*Hemo*TPS *(GenBank accession no. EU679406) consisting of 755 amino acid encodes a putative *TPS *and a *TPP *domain in tandem. Phylogenic tree analysis of multiple sequence alignments of *TPS *gene revealed that *C. sapidus *Hemocytes *TPS *(*Cas*Hemo*TPS*) is closely related to those of insects, forming a separate group from *E. coli, Saccharomyces cerevisae*, and *Ulva prolifera *(Fig. 1) [19]. The *TPS *gene in arthropods appears to be a fused gene of a homolog of *Ost *A and *Ost *B in *E. coli*.

### Spatial distribution of TPS gene expression in various tissues of *C. sapidus*

**Figure 2:**
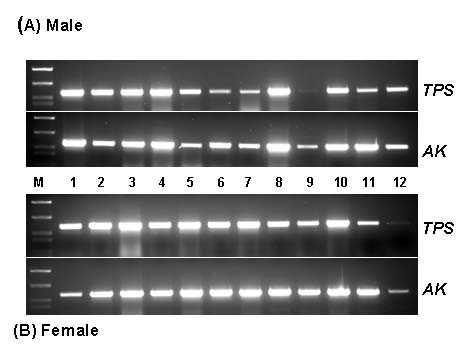
Spatial distribution of *TPS *gene in cDNAs of various tissues of adult male and female of *C. sapidus*. TPSF2 and TPSR1 primers as listed in Table 1 was used for PCR amplification with 12.5 ng of total RNA equivalent of each tissue. Arginine kinase served for a reference gene. Lane 1: eyestalk, 2: brain, 3: thoracic ganglion, 4: antennal gland, 5: gill, 6: hindgut, 7: heart, 8: chelae muscle, 9: hypodermis, 10: testis (A) and ovary (B), 11: hepatopancreas, and 12: Y-organ.

cDNAs of various tissues prepared from an adult male and female *C. sapidus *were tested for the *TPS *expression. As shown in Fig. 2, *TPS *expression was ubiquitous in all the tissues of both sexes of adult crabs, indicating that all these tissues could produce trehalose. It appears that multiple isoforms of *TPS *genes are present in tissues of the blue crab, as three of these, coding both *TPS *and *TPP*, have already been identified (unpublished observation). This wide distribution of *TPS *gene in crab tissues is surprising in contrast to what has been described in insects. In insects, the fat body is known as the exclusive biosynthetic site of trehalose [4,20,21]. After synthesis in the fat body, trehalose is released into hemolymph and serves as a major hemolymph sugar for energy required during flight.

### The effect of LPS on the expression of *TPS*, *TPS *activity and trehalose levels

**Figure 3:**
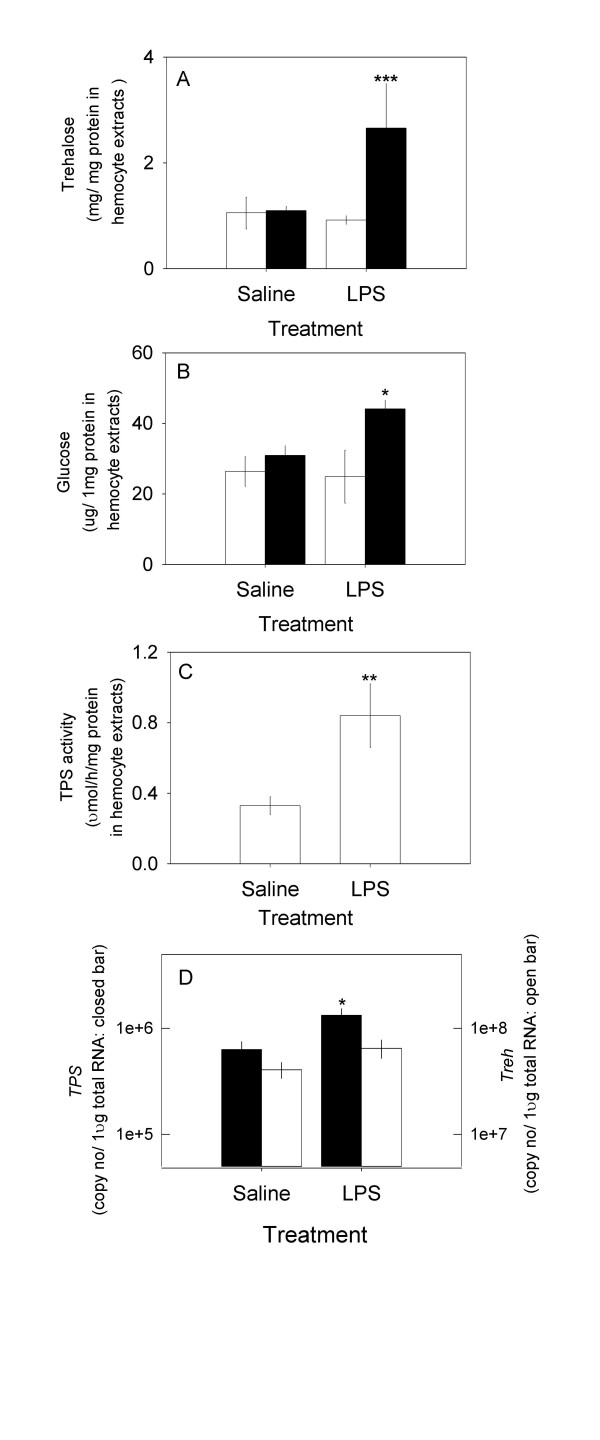
The effects of LPS injection after 24 h on the levels of intracellular glucose, trehalose, TPS enzyme activity, and the expressions of *TPS *and *Treh *in hemocytes. A) Trehalose: open bar at t = 0 h, solid bar at 24 h; B) Glucose: open bar at t = 0 h, solid bar at 24 h; C) TPS enzyme activity at 24 h; and D) expressions of *TPS *and *Treh *at 24 h: solid bar = *TPS*; open bar = *Treh*. Data is presented as mean ± 1 SE (n = 5–8) of trehalose in mg/mg protein in hemocyte extracts, of glucose in μg/mg protein in hemocyte extracts, of TPS enzyme activity in μmol/h/mg protein in hemocyte extracts and *TPS *and *Treh *expressions in copy number/μg total RNA. Statistical significance at P < 0.05 = *, at P < 0.05 = **, at P < 0.001 = ***.

Animals were challenged by the injection of 1 μg LPS to test the response of trehalose. The resting level of trehalose in hemocytes was higher than in hemolymph: 3.5 ± 0.3 mg (n = 6) (Fig. 3A) and 1.1 ± 0.1 mg/ml (n = 6), respectively. In contrast, the level of glucose was higher in hemolymph than in hemocytes: 180 ± 14.6 μg/ml (n = 6) and 70 ± 10 μg/mg protein in hemocyte extracts (n = 6), respectively (Fig. 3B). Overall, the concentration of trehalose was higher than glucose in both hemolymph and hemocytes: 6 and 50 fold, respectively, suggesting that trehalose is a major sugar in crab hemolymph as in insects [4,21]. The intracellular level of trehalose was increased ~2.5 fold in response to the LPS challenge, while a modest 1.5 fold elevation of glucose was found. LPS injection after 24 h did not cause general hypertrehalosemia or hyperglycemia in hemolymph in *C. sapidus*, although it was reported that a much higher dose of LPS induced hyperglycemia after 2 h in other crustacean species [13,15]. LPS induced a significant 2.5 fold increase in Hemo*TPS mRNA*, a three fold increment of TPS activity, compared to those of the controls (Figs. 3C and 3D). This could be responsible for the increase in trehalose levels in Fig. 3A. A slight change (130%) in the level of *trehalase (Treh) *mRNA that breaks down trehalose into two glucose molecules is responsible for the modest rise (1.5 fold) in intracellular glucose. The basal level of *TPS mRNA *in hemocytes was ~100 fold less than that of *Treh*.

Our result demonstrates that hemocytes possess *TSP *and *Treh *for the synthesis and metabolism of trehalose. More importantly, they modulate cellular trehalose levels for physiological and biochemical adaptation under LPS challenge, through the dynamic regulation of the expression of *TPS *and TPS enzyme activity. Furthermore, our data indicate that *C. sapidus *expresses *TPS *in multiple tissues, in contrast to insects where the fat body is considered the exclusive biosynthesis site of this sugar. Considering trehalose is the major blood sugar, it is also likely to be involved in crustacean hyperglycemia. We anticipate its ubiquitous presence in most if not all crustacean hemolymph with similar functions as those found in insects.

### Levels of TPS activity in hemocytes and trehalose in hemolymph during a molt cycle

**Figure 4:**
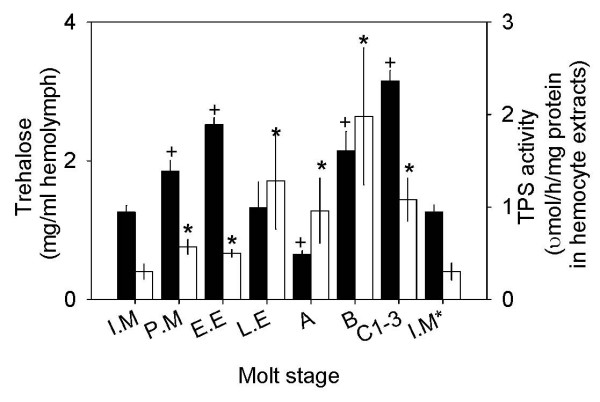
Changes in the levels of trehalose in hemolymph and TPS activity in hemocytes during molt cycle. Data is presented as mean ± 1 SE (n = 8–15) of trehalose in mg/ml hemolymph (solid bar) and of TPS enzyme in μmol/h/mg protein in hemocyte extracts (open bar). Statistical significances at P < 0.05 marked as + (trehalose) or * (TPS activity) were determined using Student's *t *test by comparing the values at intermolt stage with each different molt stage. I.M = intermolt, P.M = premolt, E.E = early ecdysis, L.E = late ecdysis, A = within 3 h after ecdysis, B = 1–2 days after ecdysis, C_1–3 _= 3–7 days after ecdysis, and I.M* = same as I.M.

Concentrations of trehalose in hemolymph of *C. sapidus *showed a bimodal pattern that exhibited two peaks during molt cycle, at early ecdysis and post ecdysis C_1–3 _(Fig. 4). The lowest level of trehalose (0.65 ± 0.05 mg/ml hemolymph, n = 8) was measured at stage A during and after the occurrence of the largest water intake occurred [22,23]. The fluctuation of TPS activity in hemocytes was also noted during the molt cycle from the lowest at intermolt to the highest at postmolt stage B: 0.3 ± 0.08 μmol/h/mg protein in hemocyte extracts (n = 12) and 1.98 ± 0.74 μmol/h/mg protein in hemocyte extracts (n = 7), respectively. TPP enzyme activity of HemoTPS was determined only at intermolt by measuring [Pi] in the same samples that were prepared for TPS activity. The activity of TPP was slightly high: 0.78 ± 0.41 μmol [Pi]/h/mg protein in hemocyte extracts (n = 5), however, this value was not significantly different from that of TPS activity. In general, TPS activity was elevated at premolt and peaked at stage B, which correlates with the highest concentration of trehalose noted at stage C_1–3_. The level of trehalose and TPS activity at the postmolt stage imply a possible involvement of this sugar in chitin synthesis, as found in insects [24]. Chitin synthesis is required for cuticle hardening and the calcification process in the exoskeleton of animals after ecdysis.

## Conclusion

We isolated, for the first time in crustaceans, the cDNA sequence of the *TPS *gene coding functional and active domains of TPS and TPP in hemocytes of *C. sapidus *where its expression was widespread in most tissues. LPS injection into animals, mimicking the induction of internal stress, stimulated the expression and enzyme activity of TPS in hemocytes, resulting in the increase in intracellular trehalose in hemocytes. Our results provide evidence of the presence and a possible adaptive function of trehalose in energy metabolism and stress response of decapod crustaceans.

## Materials and methods

### Animals

Juvenile blue crabs, *C. sapidus *(20–30 mm carapace width), were received from the blue crab hatchery in the Aquaculture Research Center, Center of Marine Biotechnology (University of Maryland Biotechnology Institute, Baltimore, MD) and reared as described [25].

### 5', 3' RACEs of *C. sapidus TPS *gene

Hemocytes were harvested from 1 ml hemolymph withdrawn in a sterilized marine anticoagulant (filtered through 0.22 μm membrane) at 1:1 ratio and immediately spun at 800 g for 10 min 4°C. After discarding the plasma, the pelleted cells were washed once in 100 μl of anticoagulant and re-centrifuged as above. The washed hemocytes were homogenized and total RNA extraction and quantification were carried out by following the procedures as described [26]. Degenerate primers of *TPS *were generated based on the conserved region of insect genes listed in GenBank using a multiple alignment program, CLUSTALW http://www.genome.jp).

The synthesis of 3' RACE cDNA of total RNA of hemocytes was carried out using GeneRacer™ (Invitrogen), while 5' RACE cDNAs was produced using SMART cDNA synthesis kit (BD Biosciences). Touchdown PCR was employed for initial amplification of *TPS*: dF1 (5'TTYGAYTCYTAYTAYAAYGG3') and dR1 (5'TCDCCRGCDCCRGCRAADGG3'). The cDNA was amplified with Advantage Taq polymerase (BD Biosciences) at the following PCR conditions: after initial denaturation for 2.5 min at 94°C, 3 cycles each step at annealing temperatures: 47°C, 45°C, and 43°C and the final step at 48°C for 25 cycles. The final amplification was achieved at annealing temperature 48°C. The touchdown PCR products served as templates for the nested PCR of TPS with a primer combination of dF2 (5' TTYTGGCCNYTNTTYCAYTCYATGCC 3') and dR2 (5'ATYTGRCARGCSACRAAYTC3') at 55°C annealing temperature. For the *TPS *gene, the cDNA from hemocytes produced a band with an expected size of 900 bp. The cloning and sequencing procedures were as stated [27]. Based on the obtained *C. sapidus *sequences of *TSP*, the following gene specific primers (listed in table 1) were made for the completion of 5', 3' RACE.

**Table 1:** The list of primer sequences that was used for cloning of *TPS *gene and QRT-PCR

	Primer sequences (5'-3')
TPS dR1	TCDCCRGCDCCRGCRAADGG
TPS dR2	ATYTGRCARGCSACRAAYTC
TPS 3dF1	TTYGAYTCYTAYTAYAAYGG
TPS 3dF2	TTYTGGCCNYTNTTYCAYTCYATGCC
TPSF1	ATGCCTGACAGAGCAACATTTCAG
TPSF2(=QF1)	ATGTTGGTGGAACACAATTCAAGGAC
TPSR1	TACAGAAGAGTCTCGGTAGAATGCA
TPSR2(=QR)	CTTTGTATAATCTAACCGATCCACTC
TPSR3	GCACGGAGTCTGGGTGGCTCTCA

'd' represents degenerate primers. Two forward primers of TPS F1 and F2 were used for 3' RACE and three of TPSR1, R2, and R3 were for 5'RACE. Primers of QF1 and QR1 were used for QRT-PCR analysis.

### Spatial distribution of TPS in various tissues of *C. sapidus*

Tissues were collected from male and female crabs at intermolt stage after they were anesthetized on ice as follows: eyestalk, brain, thoracic ganglion, antennal gland, gill, hindgut, heart, chelae muscle, hypodermis, testis or ovary, hepatopancreas, and Y-organ. Total RNAs were extracted using TRIzol^® ^(Invitrogen) and quantified with a NanoDrop 1000 (Thermo Scientific). After treatment with DNase I to eliminate genomic DNA contamination, one μg of total RNAs were used for the first cDNA synthesis with MMLV and random hexamers (Promega). Samples of cDNAs (each 12.5 ng) were amplified with a combination of primers: forward, 5'ATGTTGGTGGAACACAATTCAAGGAC3' and reverse, 5' TACAGAAGAGTCTCGGTAGAATGCA for TPS. Arginine kinase, a reference gene, was amplified using the same primers as described [27]. The PCR conditions were as follows: initial denaturation at 94°C for 2.5 min, 35 cycles at 94°C for 20 sec, 60°C for 20 sec, 70°C for 30 sec sec, and final step at 70°C for 5 min. PCR products were visualized by staining with ethidium bromide after electrophoresis on a 1.5% agarose gel.

### Lipopolysaccharide challenge

Prior to the injection of LPS or saline, 100 μl of hemolymph was withdrawn from juvenile animals (70–90 mm, carapace width) as described [27] to establish the resting levels of glucose and trehalose in hemocytes. Animals in the test group received 1 μg LPS (*E. coli *0111:B4, Sigma) in 100 μl crustacean saline, while control animals received 100 μl saline alone. 24 h after injection, 500 μl of hemolymph were withdrawn in an anticoagulant at a ratio of 1:1 and immediately centrifuged as described above. The hemocytes were re-suspended in ice cold DEPC treated PBS or Tris buffered saline and homogenized. Half of the samples were dedicated for estimating glucose, trehalose, and TPS activity, while the rest were used for RNA extraction as described above. Hemocyte protein was determined using BioRad DC protein assay (BioRad).

### Quantitative RT-PCR analysis (QRT-PCR)

The extraction and quantification procedures of total RNA of hemocytes and cDNA synthesis were stated in Chung and Zmora [27]. Standards for QRT-PCR were produced as described [25]. Sample cDNAs (12.5 – 25 ng) were analyzed for the estimation of the expressions of *TPS *using primers of QF: 5' ATGTTGGTGGAACACAATTCAAGGAC3' and QR: 5' CTTTGTATAATCTAACCGATCCACTC3' and the data were calculated as copy number/μg of total RNA of hemocytes. The level of hemocyte *trehalase *(*Treh*, GenBank accession no. EU679407) was quantified using the following primers, QF: 5' GCAGAGAGTGGATGGGA3' and QR: 5' CCCTGACAGCAGCAAGCCCTCA3'. The expression levels of *TPS *and *Treh *were represented as copy number/μg total RNA as described [26].

### Estimation of glucose and trehalose in hemocytes

Glucose levels in hemocytes were determined using glucose oxidase/peroxidase assay (Sigma) as described [28]. Trehalose concentration in hemocytes was estimated by subtracting the amount of glucose from the values determined by anthrone assay, as this assay measures both sugars [29]. Trehalose (Sigma) was used for the standard of anthrone assay. The results were presented as μg glucose or mg trehalose/mg protein in hemocyte extracts.

### Two-step TPS activity assay

TPS activity in hemocytes was estimated using a modified procedure that was previously described [30,31]. For the first step of the synthesis of trehalose 6-phospate, 100 μg of extracts from hemocytes was incubated in 200 μl final volume of the first reaction mixture containing 50 mM HEPES buffer (pH 7.1), 5 mM UDP-glucose (UDPG), 10 mM glucose-6-phosphate, and 12.5 mM MgCl_2 _at 35°C for 30 min. In controls, glucose-6-phosphate was omitted. The reactions were terminated with heat treatment at 100°C for 5 min and were centrifuged at 13,000 rpm for 5 min at room temperature. For the second step, the supernatants (150 μl) were further incubated at 35°C for 10 min in the following reaction mixture (150 mM HEPES buffer, pH 7.6, 2 mM phosphoenolpyruvate, 0.5 mM NADH, 5 U lactic dehydrogenase and 5 U pyruvate kinase). Samples were cooled on ice for 5 min and briefly centrifuged for 13,000 rpm for 1 min. 100 μl of the supernatant was placed into a 96 well plate, and the absorbance was measured at 340 nm (Spectra M5, Molecular Device). Known concentrations of UDP at 1000, 500, 250, 125, and 62.5 nmol were treated as above and served for a standard curve. TPS activity was calculated per μmol UDP/h/mg hemocyte protein.

### Trehalose 6-phosphate phosphatase (TPP) assay

In order to test the functionality of TPP domain of CasHemo*TPS*, the hemocytes were extracted in Tris-buffered saline and TPP enzyme activity was measured by following the procedure described in Klutts *et al*. [32]. The activity was calculated as μmol [Pi]/h/mg hemocyte protein.

### Estimation of TPS activity in hemocytes during molt cycle

Hemolymph samples were collected from animals at molt stages as described [33] and assayed as described above. Hemocytes homogenized in 200 μl of ice cold PBS by sonication (Branson); the extracts were centrifuged at 14,000 rpm for 10 min at 4°C; and, the supernatants were collected for the estimation of protein concentration as described above.

### Statistical analysis

Statistical significance was determined at P < 0.05 using GraphPad InStat 3 program (GraphPad Software, Inc).

## Abbreviations

TPS: trehalose 6-phosphate synthase gene; Treh: trehalase

## Competing interests

The author declares that they have no competing interests.

## Authors' contributions

JSC carried out the molecular cloning of TPS gene, TPS and TPP bioassays, and the bioassays.

## Acknowledgements

JSC thanks O. Zmora and the personnel in the blue crab hatchery program for the juvenile crabs and S. Rogers and the ARC personnel for maintaining the water quality of the re-circulation system. This article is contribution no. 08-193 of the Center of Marine Biotechnology (University of Maryland Biotechnology Institute, Baltimore, MD, USA), and the work is supported by a program grant (NA17FU2841) from NOAA Chesapeake Bay Office to the Blue Crab Advanced Research Consortium.

## References

[B1] Clegg JS The origin of trehalose and its significance during the formation of encysted dormant embryos of *Artemina salina* Comparative Biochemistry and Physiology 1965 14 135 143 10.1016/0010-406X(65)90014-9 14288194

[B2] Goyal K Browne JA Burnell AM Tunnacliffe A Dehydration-induced tps gene transcripts from an anhydrobiotic nematode contain novel spliced leaders and encode atypical GT-20 family proteins Biochemie 2005 87 565 574 10.1016/j.biochi.2005.01.010 15935281

[B3] Sakurai M Furuki T Akao A Tanaka D Nakahara Y Kikawada T Watanabe M Okuda T Vitrification is essential for anhydrobiosis in an African chironomid, *Polypedilum vanderplanki* Proceedings of the National Academy of Sciences USA 2008 105 5093 5098 10.1073/pnas.0706197105 PMC2278217 18362351

[B4] Becker A Schloder P Steele JE Wegener G The regulation of trehalose metabolism in insects Experientia 1996 52 5 433 439 10.1007/BF01919312 8706810

[B5] Wyatt GR Kalf GF The chemistry of insect hemolymph. Trehalose and other carbohydrates Journal of General Physiology 1957 40 833 846 10.1085/jgp.40.6.833 13439163 PMC2147581

[B6] Zoltowska K Lopieniska-Biernat E Content of glycogen and trehalose and activity of α-amylase and trehalase in *Galleria mellonella *larvae infected with entomophthogenic nematodes *Steinemema affinis *and *S. feltiae* Wiad Parazytol 2006 52 2 103 107 17120991

[B7] Chen Q Ma E Behar KL Xu T Haddad GG Role trehalose phosphate synthase in anoxia tolerance and development in *Drosophila melanogaster* Journal of Biological Chemistry 2002 277 3274 3279 10.1074/jbc.M109479200 11719513

[B8] Chang ES Keller R Chang SA Quantification of crustacean hyperglycemic hormone by ELISA in hemolymph of the lobster, *Homarus americanus*, following various stresses General and Comparative Endocrinology 1998 111 359 366 10.1006/gcen.1998.7120 9707481

[B9] Chung JS Webster SG Dynamics of *in vivo *release of molt-inhibiting hormone and crustacean hyperglycemic hormone in the shore crab, *Carcinus maenas* Endocrinology 2005 146 5545 5551 10.1210/en.2005-0859 16150903

[B10] Johnston MA Spencer Davies P Elder HY Possible hepatic function for crustacean blood cells Nature 1971 330 471 472 10.1038/230471a0 4929983

[B11] Keller R Andrew EM The site of action of the crustacean hyperglycemic hormone General and Comparative Endocrinology 1973 20 572 578 10.1016/0016-6480(73)90089-0 4715238

[B12] Lorenzon S Edomi P Giulianini PG Mettulio R Ferrero EA Variation of crustacean hyperglycemic hormone (cHH) level in the eyestalk and haemolymph of the shrimp *Palaemon elegans *following stress Journal of Experimental Biology 2004 207 4205 4213 10.1242/jeb.01264 15531641

[B13] Lorenzon S Giulianini PG Ferrero EA Lipopolysaccharide-induced hyperglycemia is mediated by CHH release in crustaceans General and Comparative Endocrinology 1997 108 395 405 10.1006/gcen.1997.6986 9405116

[B14] Sedlmeier D The role of hepatopancreatic glycogen in the action of the crustacean hyperglycemic hormone (CHH) Comparative Biochemistry and Physiology 1987 87a 423 435

[B15] Stentiford GD Chang ES Chang SA Neil DM Carbohydrate dynamics and the crustacean hyperglycemic hormone (CHH) effects of parasitic infection in Norway lobsters (*Nephrops norvegicus*) General and Comparative Endocrinology 2001 121 13 22 10.1006/gcen.2000.7575 11161766

[B16] Issartel J Renault D Voituron Y Bouchereau A Vernon P Hervant F Metabolic responses to cold in subterrranean crustaceans Journal of Experimental Biology 2005 208 2923 2929 10.1242/jeb.01737 16043597

[B17] Siebers D Lucu C Sperling K-R Eberlein K Kinetics of osmoregulation in the crab *Carcinus maenas* Marine Biology 1972 17 291 303 10.1007/BF00366739

[B18] Lipcius RN Seitz RD Seebo MS Colon-Carrion D Density, abundance and survival of the blue crab in seagrass and unstructural salt marsh nurseries of Chesapeake Bay Journal of Experimental Marine Biology and Ecology 2005 319 69 80 10.1016/j.jembe.2004.12.034

[B19] Dereeper A Guignon V Blanc G Audic S Buffet S Chevenet F Dufayard JF Guindon S Lefort V Lescot M Phyologeny.fr: robust phylogenetic analysis for the non-specialist Nucleic Acids Research 2008 36 W465 459 10.1093/nar/gkn180 18424797 PMC2447785

[B20] Mitsumasu K Azuma M Niimi T Tamashita O Yaginuma T Membrane-penetrating trehalase from silkworm *Bombyx mori*. Molecular cloning and localization in larval midgut Insect Molecular Biology 2005 14 501 508 10.1111/j.1365-2583.2005.00581.x 16164606

[B21] Waytt GR Kalf GF The chemistry of insect hemolymph. Trehalose and other carbohydrates Journal of General Physiology 1957 40 833 846 10.1085/jgp.40.6.833 13439163 PMC2147581

[B22] Chung JS Dircksen H Webster SG A remarkable, precisely timed release of hyperglycemic hormone from endocrine cells in the guts is associated with ecdysis in the green crab, *Carcinus maenas* Proceedings of the National Academy of Sciences USA 1999 96 13103 13107 10.1073/pnas.96.23.13103 PMC23907 10557280

[B23] Neufeld DS Cameron JN Mechanism of the net water uptake in molting blue crabs (*Callinectes sapidus*) acclimated to high and low salinities Journal of Experimental Biology 1994 188 11 23 9317238 10.1242/jeb.188.1.11

[B24] Tang B Chen X Liu Y Tian H Liu J Hu J Xu W Zhang W Characterization and expression patterns of a membrane-bound trehalase from *Spodoptera exigua* BMC Molecular Biology 2008 9 51 10.1186/1471-2199-9-51 18492231 PMC2424068

[B25] Chung JS Webster SG Moult cycle-related changes in biological activity of moult-inhibiting hormone (MIH) and crustacean hyperglycaemic hormone (CHH) in the crab, *Carcinus maenas* European Journal of Biochemistry 2003 270 3280 3288 10.1046/j.1432-1033.2003.03720.x 12869204

[B26] Chung JS Wilcockson DC Zmora N Zohar Y Dircksen H Webster SG Identification and developmental expression of mRNA encoding crustacean cardioactive peptide (CCAP) in decapod crustaceans Journal of Experimental Biology 2006 209 3862 3872 10.1242/jeb.02425 16985202

[B27] Chung JS Zmora N Functional studies of crustacean hyperglycemic hormone (CHHs) of the blue crab, *Callinectes sapidus *– the expression and release of CHH in eyestalk and pericardial organ in response to environmental stress FEBS J 2008 275 4 693 704 10.1111/j.1742-4658.2007.06231.x 18190527

[B28] Webster SG Measurement of crustacean hyperglycaemic hormone levels in the edible crab *Cancer pagurus *during emersion stress Journal of Experimental Biology 1996 199 1579 1585 9319482 10.1242/jeb.199.7.1579

[B29] Roe JH The determination of sugar in blood and sinal fluid with anthrone reagent Journal of Biological Chemistry 1955 212 335 343 13233235

[B30] Hottiger T Schmutz P Wiemken A Heat-induced accumulation and futile cycling of trehalose in Saccharomyces cerevisiae Journal of Bacteriology 1987 169 5518 5522 2960663 10.1128/jb.169.12.5518-5522.1987 PMC213980

[B31] Valenzuela-Soto EM Marquez-Escalante JA Iturriaga G Figuerosa-Soto CG Trehalose 6-phosphate synthase from *Selaginella lepidophylla*: purification and properties Biochemical and Biophysical Research Communications 2004 313 314 319 10.1016/j.bbrc.2003.11.128 14684162

[B32] Klutts S Pastuszak I Koroth V Edavana K Thampi P Pan YT Abraham EC Carroll D Elbein AD Purification, cloning, expression, and properties of Mycobacterial trehalose-phosphate phosphatase Journal of Biological Chemistry 2003 278 2093 2100 10.1074/jbc.M209937200 12417583

[B33] Drach P Tchernigovtzeff C Sur la methode de determination des stades d'intermue et son application-generale aux Crustaces Vie et milieu series A Biol 1967 18 595 610

